# A unifying mathematical framework for experimental TCR-pMHC kinetic constants

**DOI:** 10.1038/srep46741

**Published:** 2017-04-26

**Authors:** Jose Faro, Mario Castro, Carmen Molina-París

**Affiliations:** 1 Area of Immunology, Faculty of Biology, and Biomedical Research Center (CINBIO), Universidade de Vigo, Vigo, Spain; 2 Instituto Gulbenkian de Ciência, Oeiras, Portugal; 3 Grupo Interdisciplinar de Sistemas Complejos (GISC) and DNL, Universidad Pontificia Comillas, Madrid E-28015, Spain; 4 Department of Applied Mathematics, School of Mathematics, University of Leeds, Leeds, LS2 9JT, UK

## Abstract

Receptor binding and triggering are central in Immunology as T cells activated through their T cell receptors (TCR) by protein antigens orchestrate immune responses. In order to understand receptor-ligand interactions, many groups working with different experimental techniques and assays have generated a vast body of knowledge during the last decades. However, in recent years a type of assays, referred to as *two*-*dimensional* or membrane-to-membrane, has questioned our current understanding of the role of different kinetic constants (for instance, *on*- versus *off*-rate constants) on TCR-ligand interaction and subsequent T cell activation. Here we present a general mathematical framework that provides a unifying umbrella to relate fundamental and effective (or experimentally determined) kinetic constants, as well as describe and compare state-of-the-art experimental methods. Our framework is able to predict the correlations between functional output, such as 1/*EC*_50_, and effective kinetic constants for a range of different experimental assays (in two and three dimensions). Furthermore, our approach can be applied beyond Immunology, and serve as a “translation method” for the biochemical characterization of receptor-ligand interactions.

T lymphocytes and their activation through T cell receptors (TCR) are essential components of most antigen-specific immune responses and their regulation. The cellular outcome of the interaction between a TCR and its ligand (a peptide bound to a major histocompatibility complex molecule [pMHC]) depends on their binding strength. However, how to quantitatively define this strength has proven a difficult task.

Technical developments in the last twenty years have notably increased our quantitative knowledge of the kinetics of pMHC recognition by T lymphocytes. Traditional assays for assessing TCR-pMHC interaction strength in terms of rate or affinity constants are designed such that TCRs or pMHC ligands are placed in *solution* (three-dimensional [3D] assays)^1^,^2^,^3^,^4^,^5^,^6^. However, classification of T cell ligands according to their 3D strength of interaction with TCRs was often inconsistent with their *potency* (level of *functional* output on T cells)^1^,^7^,^8^,^9^. To account for these discrepancies, it has been suggested that although 2D off-rates in the membrane environment could be estimated from solution measurements using a confinement time model^7^, ultimately direct measurements of *in situ* TCR-pMHC interaction kinetics would be required.

Two papers published in 2010^10^,^11^ revealed essential differences between traditional assays and those in which TCRs and their pMHC ligands are confined to a membrane (referred to as 2D assays). These studies have, thus, dramatically highlighted the impact of dimensionality on the kinetics of reversible protein-protein interactions. For instance, adhesion frequency and thermal fluctuations assays^10^ showed not only significantly different estimations of kinetic parameters, such as the affinity constant or the off-rate constant but, perhaps more importantly, qualitative discrepancies in the classification of ligands according to their potency. This is summarized in Fig. 1, where we provide a comprehensive comparison of kinetic constants in 2D and 3D. This figure generalises that of ref. 12, as it provides a comprehensive account of the correlation between kinetic constants and the functional output of T cells (as measured by the inverse of the effective pMHC concentration stimulating half-maximal T cell proliferation, 1/*EC*_50_).

Figure 1 shows that the correlation between 1/*EC*_50_ (functional output) and kinetic constants is the opposite in 2D to that of 3D (based on refs 8,10). Similar conclusions were made by other authors using single-molecule fluorescence resonance energy transfer (FRET) microscopy at the cell level^11^. Thus, 2D *on*-rate constants appear to be, ultimately, responsible for the differences in ligand potency, suggesting that 2D experiments are mandatory in order to understand TCR-mediated T cell activation. This contradicts the (3D) previously held view that the *off*-rate constant was the main discriminant between ligands, given a fixed TCR, to assess their potency.

It is then timely to ask whether or not it is possible to reconcile 2D and 3D experimental observations, and thus, the large body of knowledge accumulated during the last decades on effective kinetic parameters of TCR-pMHC interactions. Furthermore, cellular decisions (proliferation, differentiation, migration, or death) are regulated by reversible protein-protein interactions, either in 2D or 3D scenarios. Thus, an answer to the previous question has important practical implications in Cell Biology.

In this paper, we approach this problem as follows. First, we develop a general mathematical framework, based on ordinary differential equations, to describe the different intermediate processes and chemical species involved in the specific binding of a TCR to its ligand in terms of fundamental constants for translational and rotational diffusion, as well as chemical binding. Then, based on a comprehensive analysis of current experimental assays, we identify which processes (translational diffusion, rotational diffusion or chemical binding) or species cannot be observed in each assay. In this way, we are able to relate fundamental and effective (or experimentally determined) kinetic constants. Thus, our mathematical approach makes explicit the meaning of effective kinetic constants, as used in the different experimental settings.

In summary, the proposed framework not only allows us to help understand current experimental differences in the estimation of TCR-pMHC kinetic parameters but, also, provides a unifying umbrella to describe and compare 2D and 3D state-of-the-art experimental systems for different ligand-receptor systems.

## Results

### Qualitative description of binding dynamics

Binding dynamics in 2D and 3D involve multiple steps, which can be purely chemical in nature or constrained by the geometry of the experiment. We can recognize three steps^13^,^14^,^15^,^16^,^17^, as depicted in Fig. 2: 1) diffusion/encounter, which is *dimension* dependent and ligand independent, 2) rotation/orientation, which is also dimension dependent (as the geometry constrains rotational degrees of freedom) and ligand independent, and 3) molecular association and dissociation, which only depends on the specific chemical properties of the interacting TCR and pMHC molecules. Only the *binding* step, or molecular complex formation, can potentially lead to an allosteric conformational change in the TCR, a possibility recently shown to occur in the TCR *β* chain^9^,^18^,^19^,^20^.

### General reaction kinetics and commonly used, specific ligand-receptor models

The qualitative picture (see Fig. 2) of the different binding steps in two and three dimensions can be expressed in terms of molecular kinetic reactions. Specifically,


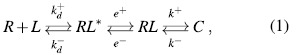


where *R* is the concentration of free receptors (TCR), *L* the concentration of free ligands (pMHC), RL* a *virtual* (encounter) complex of one *R* and one *L* molecule characterized by being within the reaction distance, *RL*, a complex state similar to RL* but with *R* and *L* suitably oriented, *i.e*., potentially allowing the binding of TCR to pMHC, and finally, *C* the concentration of the bound complex (that would, eventually, trigger in T cells a signaling cascade). The fundamental (on and off) kinetic constants introduced in Fig. 2 and the reactions (1), as well as the corresponding affinity constants, are summarized in Table 1. In particular, *k*^+^ and *k*^−^ are, respectively, the chemical association and dissociation rate constants corresponding to a protein-protein complex (docked conformation) or reversible bond formation or disruption. They are denoted here the fundamental binding rate constants. When we compare experiments with different dimensionality, the explicit notation 2D or 3D will be used.

In order to compare different experimental assays, we only need to be concerned with observables that can be quantified over long enough times or in equilibrium, and thus, we ignore stochastic effects^22^. Consequently, the mathematical model that describes the above reactions (1) can be formulated, in a *deterministic* approximation, as the following system of coupled ordinary differential equations (ODEs):


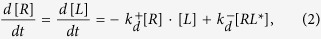



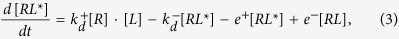



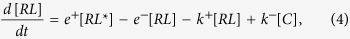



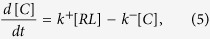


where the square brackets represent the concentration of the different molecular species.

In the Supplementary Information (SI) we discuss the main 3D and 2D experimental assays used to analyze the kinetic constants of TCR-pMHC interactions, and describe what can be measured in each assay and the chemical model used to derive the different kinetic constants. This will allow us to select the reactions and/or chemical species described by equations (2), (3), (4), (5), that are *grouped* in each experimental assay. We identify four different “effective” models. These models are described in the following and in Fig. 3, where the relation between each “effective” model and the general one has been explicitly shown.

#### Pre-binding model A (PBA)

In this model it is assumed that *encounter* and *orientation* take place in a single step, prior to molecular binding. The model groups the encounter and oriented complexes in a single chemical species that we denote by 〈*RL*〉. The rotation step is often neglected but, as shown by DeLisi^15^,^23^, it adds complexity to the frequently assumed pure diffusional mechanisms^14^,^16^. This model may correspond to the thermal fluctuation assay^10^.

#### Pre-binding model B (PBB)

In this model unbinding and disruption of orientation are assumed to take place in a single step, before the molecules diffuse away. This may also correspond to the thermal fluctuation assay^10^. This model includes FRET performed in solution (bulk FRET)^6^ and single molecule fluorescence microscopy (SMFM)^24^,^25^.

#### FRET model

The experimental system of ref. 11 allows to detect the encounter of the ligand and the receptor, but it cannot separately distinguish the initial species. Thus, if only the donor fluorescence signal is detected, this can be due to a free ligand or an encounter, non-oriented complex. On the other hand, FRET signals cannot discriminate between a bound TCR-pMHC complex and an oriented one (see Fig. 2). Mathematically, this means that we need to define *meta*-*states* composed of the original ones. Specifically, 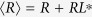, 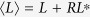 and 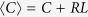. These definitions are not mathematical artefacts but, rather, represent the inherent experimental uncertainty of FRET experiments.

#### Single-step model (SS)

In this case, the experimental assay can only distinguish between two states, bound and unbound, and therefore cannot *discriminate* between translational and rotational steps. As discussed in SI, this model applies to the adhesion frequency assay and the SPR assay.

The simplifications considered in the above models involve, ultimately, the introduction of *effective kinetic constants*, corresponding to the different steps in each effective model, and summarised in Fig. 3 and Table 2. In order to study these kinetic constants, we first formulate each effective model as a set of coupled ODEs, as has been done for the general model. We then express the assumptions of each effective model in mathematical terms and apply them to the general model. Finally, the effective kinetic constants are expressed in terms of the fundamental ones (for details, see SI). Note that, as summarized in Table 2, different effective kinetic constants have different meanings and values depending on the experimental assay under consideration.

We note that while the PBA and the SS models have been widely used in the literature, as we show below, they cannot *fully* account for all the experimental observations reported in the literature of TCR-pMHC binding kinetics. We also note that the difference between the FRET and the SS models, is that the former groups together the following chemical species: *R* and RL* into 〈*R*〉, *L* and RL* into 〈*L*〉, and *RL* and *C* into 〈*C*〉, while the latter groups the three forward (reverse) reaction constants into a single effective forward (reverse) one, without the need to introduce the chemical species RL* and *RL*.

### Understanding effective constant constants derived from 2D and 3D experimental assays

The preceding section provides a “translation tool” to help identify the effective constants for each effective model in terms of the fundamental ones, as shown in Table 2, Eqs (6)–(23). This “translation” allows to classify and compare experimental observations in the literature with a unified and general mathematical framework. We now illustrate our method by considering five different cases that cover the main experimental techniques currently used. We do so by carrying out pairwise comparisons of the estimations derived from 2D and 3D experimental assays.

#### Case 1: Adhesion frequency (AF) assay *versus* thermal fluctuation (TF) assay

This case corresponds to the two different experimental assays used by Zhu and colleagues^10^ to evaluate 2D kinetics. As indicated in Table 2, there are two possible interpretations of the effective off-rate constant measured by the TF assay in ref. 10. This depends on which of the two models, PBA or PBB, applies to this method. Taking into account the observation by Zhu and colleagues that the off-rate constants of the bound complex *C* (or meta-state 〈*C*〉) estimated by the AF and the TF assays are very similar^10^, we consider both the PBA and the PBB models. In the first case, the underlying assumption is that unbinding is accompanied by an instantaneous change in ligand orientation. In the second case, we allow for multiple binding/unbinding events before the ligand changes the *optimal* orientation.

Let us explore the implications of both assumptions:

- **Pre**-**binding Model A**. In this model, the empirical observation that the AF and TF assays provide the same estimation of *k*^−^, implies that Eqs (7) and (19) should provide the same result. Hence, the following equality must hold:
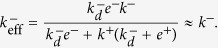
This implies that 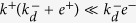 and, therefore,
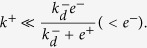
That is, the binding on-rate constant is much slower than the rotational diffusion off-rate constant.
- **Pre**-**binding Model B**. Likewise, the above constraint in this case implies the following equality, from Eqs (10) and (19):

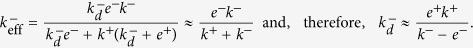


**Figure 1:**
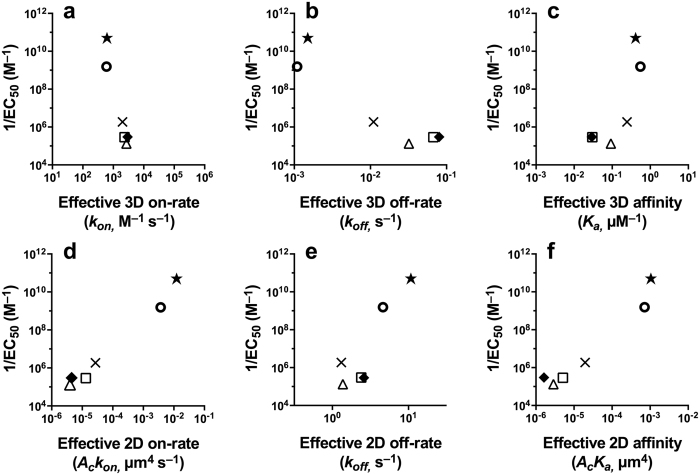
Correlation between T cell proliferation (*potency*), quantified through the value of 1/*EC*_50_, and TCR-pMHC effective kinetic parameters in 3D and 2D. (**a–c**) pMHC functional potency *versus* TCR-pMHC 3D kinetics estimated with the surface plasmon resonance assay. (**d–f**) pMHC functional potency *versus* TCR-pMHC 2D kinetics estimated with the adhesion frequency assay. The data for the top row was adapted from refs 8,10, and data for the bottom row is based on ref. 10. Symbols correspond to the following ovalbumin-derived peptides (altered peptide ligands or APLs): ★, OVA; ⚪, A2; ×, G4; 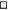, E1; 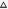, V-OVA; 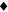, R4.

**Figure 2:**
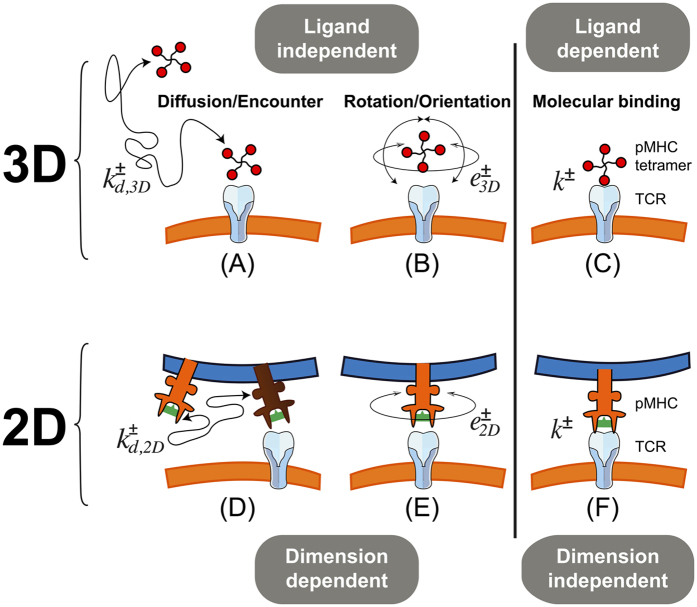
Three steps of binding in three *versus* two dimensions. Top row, binding in three dimensions: (**A**) 3D diffusion/encounter, (**B**) rotation/orientation, and (**C**) molecular binding. Bottom row, the three binding steps in two dimensions: (**D**) 2D (membrane) diffusion/encounter, (**E**) rotation/orientation, and (**F**) molecular binding. The vertical line separates dimensional dependent and independent processes. The fundamental kinetic constants corresponding to each step in 3D and 2D have been included: 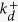, 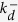 for diffusion/encounter, *e*^+^, *e*^−^ for rotation/orientation and *k*^+^, *k*^−^ for binding and unbinding, respectively. The fundamental kinetic constants are defined in Table 1.

**Figure 3:**
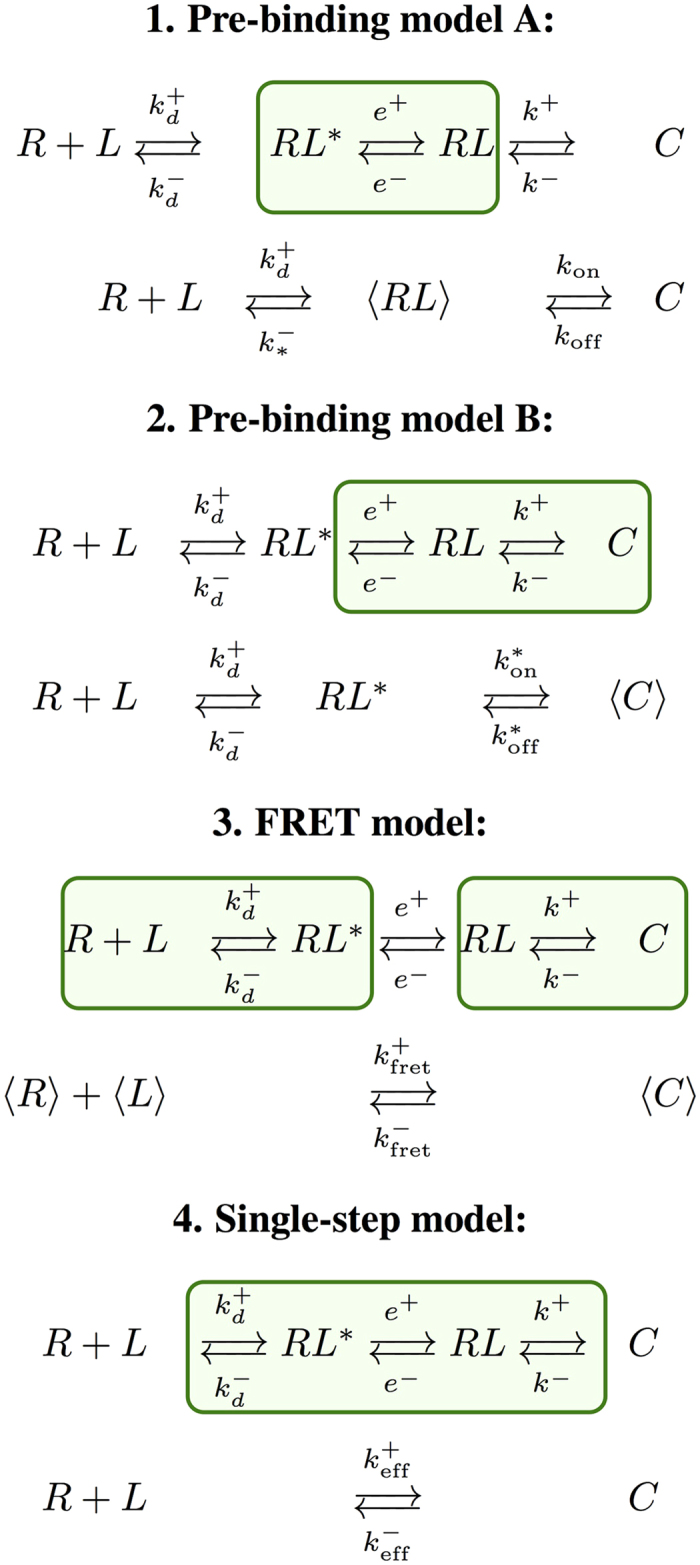
Summary of effective models. Species with *angular* brackets (〈.〉) denote effective *meta*-states. These meta-states account for the lack of microscopic details in the given experimental assay. For each model, the first row shows the full model with green boxes that emphasize the implicit *grouping* behind a given experimental assay, and the second row describes the corresponding simplified or *effective* model.

Since 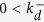, this, in turn, implies *e*^−^ < *k*^−^. That is, the rotational diffusion off-rate constant is slower than the binding off-rate constant *k*^+^. However, the constraint 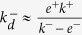 cannot hold unconditionally, as the rate constant 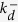 is ligand independent and *k*^+^, *k*^−^ are ligand dependent. Furthermore, the binding on- and off-rate constants change with ligand potency (for instance, compare ligands G4 and OVA in Fig. 1d,e), respectively. All this makes the PBB model unsuitable for describing TF assays.

We conclude that the TF assay is appropriately described by the PBA model, in which case, and as shown above:


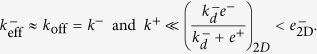


The expression on the left implies that both the TF and the AF assays are suitable experimental tools to extract the fundamental binding off-rate constant *k*^−^.

Once the correct model describing the TF assay has been identified, we observe that 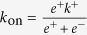 (see Eq. 10 in SI). That is, the effective on-rate constant *k*_0*n*_ is directly proportional to the fundamental binding on-rate constant *k*^+^, with a ligand-independent proportionality factor *E*^2D^/(1+*E*^2D^). However, as computed in ref. 14, *E*^2*D*^ ≈ 0.04 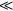 1, therefore, 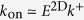 (Eq. 6 in Table 2).

#### Case 2: AF assay *versus* surface plasmon resonance (SPR) assay

Case 1 showed how the analysis of the experimental data in the light of our mathematical description (PBA model for the TF assay, and 2D SS model for the AF assay), provides a consistent picture of the underlying fundamental processes, namely, transport, orientation and binding. In a similar vein, in this and the following subsections we analyze and compare the effective kinetic parameters obtained with different techniques involving the same or different dimensionality when making use of effective models.

This second case corresponds to the approach followed by Zhu and colleagues in ref. 10 to compare 2D and 3D kinetic parameters obtained, respectively, by the AF assay and the SPR assay^1^,^26^,^27^,^28^.

Since the kinetic molecular processes in these two experimental assays can be described by the same model, namely, the SS model, the effective on- and off-rate constants determined with both assays can be written in terms of identical equations (see Table 2). Furthermore, in both assays the effective on-rate constants range from 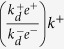 to 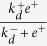, and the effective off-rate constants from *k*^−^ to 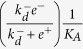.

However, these two assays differ in both the diffusion and orientation rate constants. In 2D assays the diffusion on-rate constant (units of 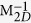
s^−1^ ≡ *μ*m^2^ mol^−1^ s^−1^) is given by 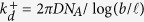, where *D* is the 2D diffusion coefficient, *N*_*A*_ Avogadro’s number, *b* the mean distance between TCR molecules on the cell membrane, and 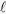 the encounter radius (of the order of the diameter of a TCR)^16^. In contrast, in the SPR assay, we have 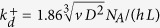, where *v* is the flow rate, *h* the height of the flow cell, and *L* the distance from the flow cell inset to the sampling area^29^. Therefore, the effective affinity constants obtained by the AF assay and the SPR assay, respectively, 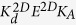 and 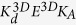, are different (in general) and, hence, their ratio is not one. Finally, as diffusion rate constants can be calculated from first principles, we can make use of experimental data to compute the ratio of the rotational diffusion affinity constants in 2D and 3D.

As shown above in Case 1, in the AF assay 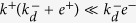, hence it follows:


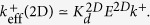


In contrast, in SPR experiments the effective 3D on-rate constant is almost independent of the ligand potency (see Fig. 1a) and, hence, it is independent of the binding constants. This experimental observation applied to the 3D SS model (Table 2) implies that 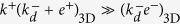, and hence:


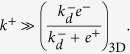


This relation implies, in turn, that 
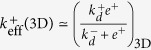
 and 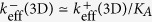.

In summary, our analysis of Cases 1 and 2, based on the experiments reported by Zhu and colleagues^10^, leads to:


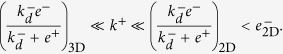


which allows to deduce how the different effective 2D and 3D parameters in the SS model, corresponding to the AF and SPR assays, can be expressed in terms of the fundamental kinetic constants.

The log-log plots of ligand *potency* (measured as 1/*EC*_50_ for T cell proliferation) and 3D and 2D experimental rate constants as determined, respectively, with the SPR and AF assays (Fig. 1) can now be used to deduce the expected correlations between ligand potency and the fundamental kinetic and affinity constants, *k*^+^, *k*^−^, and *K*_*A*_. To that end, we first calculate the log of each effective parameter. We obtain for the 2D SS model:


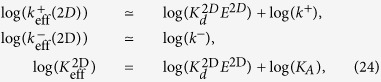


and for the 3D SS model:


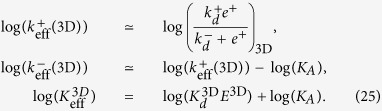


**Figure 4:**
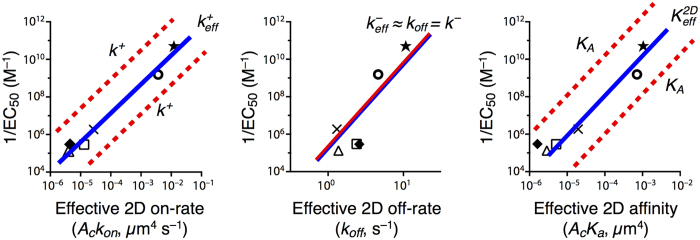
Observed and expected trend lines between T cell functional output and effective (AF assay-derived) and fundamental kinetic constants for the set of peptides used in Fig. 1. The blue solid lines correspond to the effective kinetic constants, 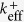, 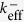, 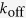 and 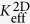, as determined with the AF assay and the SS model. The red lines correspond to the fundamental binding constants (*k*^+^, *k*^−^ and *K*_*A*_). A dashed red line indicates an uncertainty in the direction and magnitude of the shift corresponding to a given fundamental binding parameter, depending on whether 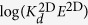 is positive or negative and whether 
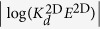
 is relatively large or small. See details in Cases 1, 2 and 4.

Now, according to the experimental results in Fig. 1d–f, in the AF assay the value of the effective 2D on- and off-rate constants and the effective affinity constant, for all peptide ligands assayed, increases with ligand potency, respectively, about a thousand-fold range, a ten-fold range, and a thousand-fold range. From Eq. (24) it follows that the fundamental kinetic parameters *k*^+^, *k*^−^, and *K*_*A*_ span the same range as 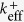, 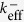, and 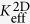, respectively. Moreover, the trend lines of the correlation between log(1/*EC*_50_) and the fundamental kinetic parameters have the same slopes as the respective ones of the effective parameters. Furthermore, except for the trend line of *k*^−^, the trend lines of *k*^+^ and *K*_*A*_ are shifted along the *x*-axis, relative to the trend lines of 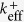 and 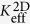, by a factor 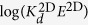. To illustrate these results, in Fig. 4 we replot Fig. 1d–f and superimpose on each panel, the trend lines corresponding to the experimentally derived parameters in the AF assay, 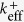, 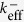, *k*_off_ and 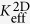 (blue lines). Also superimposed are the estimated trend lines corresponding to the fundamental kinetic constants *k*^+^, *k*^−^, *K*_*A*_ (red lines).

Strikingly, in respect to the results with the SPR assay, Eq. 25 indicates, on the one hand, that the effective on-rate constant 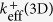 is ligand independent, and on the other hand, that the effective off-rate constant 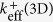 is approximately the inverse of the effective affinity constant 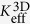 (proportional to the intrinsic binding affinity, *K*_*A*_). Not surprisingly, therefore, Fig. 1b,c shows that log(1/*EC*_50_) is negatively correlated to 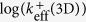 but positively correlated to 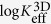.

#### Case 3: 2D fluorescent resonance energy transfer (FRET) assay *versus* SPR (3D) assay

This case corresponds to the approach followed by Davis and colleagues in ref. 11 in which the authors use 2D FRET and SPR assays to compare 2D and 3D kinetic parameters.

We have seen in Case 2 that for SPR experiments the effective on-rate constant is virtually ligand independent, and therefore 
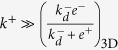
 and 
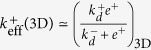
. This implies that the effective off-rate constant can be approximated by 
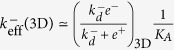
.

In the 2D FRET assay the effective on-rate constant is given by 
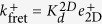
 (see Table 2) and, hence, is ligand independent. On the other hand, the effective off-rate constant, 
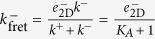
, may or may not be ligand dependent, depending on whether *K*_*A*_ is greater or lower than one. The relationship between this effective parameter and the expected ligand potency is therefore determined by the relative weights of *k*^+^ and *k*^−^. Thus, if *k*^+^ 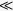 *k*^−^, the effective off-rate constant will be ligand independent. Since this is not what is experimentally observed, it follows that at most 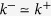. This result implies that 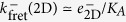.

In summary, for both 2D FRET and SPR assays the effective on-rate constants are predicted to be ligand independent (for the most part). In contrast, in both assays the effective off-rate constants are inversely proportional to the intrinsic binding affinity, *K*_*A*_, but with different proportionality factors. These dependences indicate that, experiments like those reported in Fig. 1a–c, and similar ones using the 2D FRET assay, would be misleading if wrongly interpreted as providing an approximate estimation of the fundamental binding on- and off-rate constants.

#### Case 4: 2D FRET assay *versus* AF Assay

The two laboratories pioneering these two techniques claimed that they estimate the same kinetic parameters. However, they obtained different results (see refs 10,11). As shown below, comparison between both types of 2D experiments helps to understand the origin of these discrepancies.

The determined effective on-rate constants of 2D FRET and AF assays, discussed in Cases 1 and 3, are given by Eqs (15) and (18), respectively, 
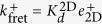
 and 
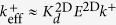
. That is, 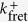 estimated in the 2D FRET assay is ligand-independent. In contrast, 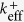 estimated in the AF assay is proportional to the fundamental binding on-rate constant, *k*^+^, with virtually the same proportionality factor for different ligands.

In the case of the effective off-rate constants of 2D FRET and AF assays, 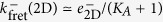 and 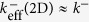 (see Table 2), both effective parameters depend on the ligand. However, there is an important difference between both assays. While in the AF assay 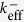 is approximately equal to the intrinsic binding off-rate constant, in the 2D FRET assay 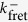 is inversely proportional to the intrinsic binding affinity constant *K*_*A*_. Therefore, it is quite possible that ligands with similar intrinsic binding off-rate constants show different effective binding off-rate constants in AF and 2D FRET assays.

In addition, the effective affinity constants also differ between both assays, with the former being given by *E*^2*D*^(*K*_*A*_ + 1) and the latter by *E*^2*D*^*K*_*A*_. Thus, they will differ considerably for *K*_*A*_ 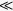 1, with the former being virtually *E*^2*D*^ for pMHC ligands having different intrinsic binding affinity constants, and the latter being directly proportional to *K*_*A*_.

**Figure 5:**
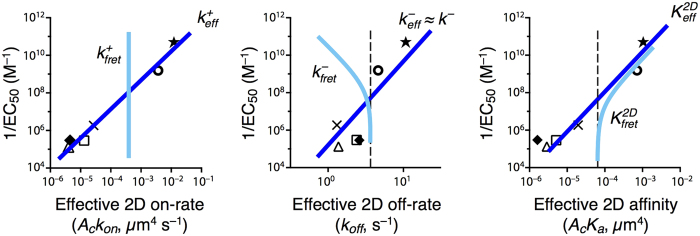
Observed and expected trend lines between T cell functional output and effective kinetic constants, derived from the 2D FRET and AF assays, for the set of peptides used in Fig. 1. The dark blue lines correspond to the effective kinetic constants 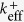, 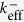, and 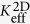 from the AF assay. The cyan lines are the predicted trend lines for the correlation between log(1/*EC*_50_) and the logarithm of the effective parameters 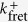, 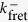, and 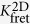 from the 2D FRET assay, as inferred using the results in Cases 1 and 3 and further discussed in Case 4. Dashed vertical lines are drawn to help visualize the asymptotic limits of 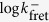 and 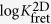. Their particular positions, as well as that of cyan lines, are for illustrative purposes.

All this is summarized schematically in Fig. 5, where Fig. 1d–f is re-plotted with trend lines superimposed, which correspond to the effective kinetic parameters obtained with the 2D FRET and AF assays. We illustrate how 2D FRET is unable to capture the fundamental kinetic constants, *k*^+^ and *k*^−^.

#### Case 5: 2D FRET *versus* 3D FRET

Finally, we compare the 2D and 3D FRET assays. According to the results summarized in Eqs (9) and (15) in Table 2, the effective binding on-rates in 2D FRET and 3D FRET (or bulk FRET) assays are 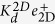 and 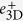, respectively. In contrast, their effective off-rates are formally similar, differing only in their corresponding dimensionality, namely, 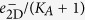 and 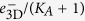. This implies that the effective on- and off-rates, and the corresponding affinity constants, differ in the 2D FRET and 3D FRET assays by the proportionality factors, 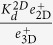, 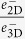, and 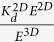, respectively. It also implies that the trend lines of the expected correlation between ligand potency and 3D FRET kinetic parameters are essentially the same than for 2D FRET kinetic parameters (see Fig. 5).

**Figure 6:**
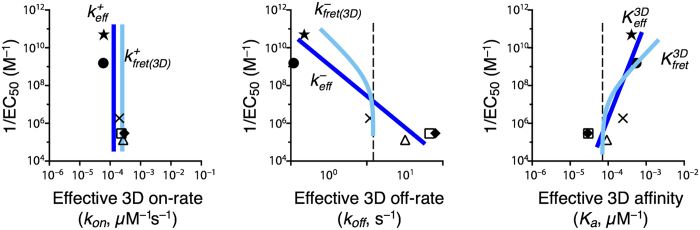
Observed and expected trend lines between T cell functional output and effective kinetic constants, derived from the 3D FRET and SPR assays, for the set of peptides used in Fig. 1. The dark blue lines correspond to the effective kinetic constants 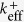, 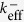, and 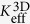 from the SPR assay. The cyan lines are the predicted trend lines for the correlation between log(1/*EC*_50_) and the logarithm of the effective parameters 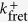, 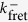, and 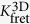 from the 3D FRET assay, as inferred using the results in Case 5. Dashed vertical lines are drawn to help visualize the asymptotic limits of 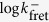 and 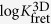. Their particular positions, as well as that of cyan lines, are for illustrative purposes.

All this is summarized schematically in Fig. 6, where Fig. 1a–c is re-plotted with trend lines superimposed, which correspond to the effective kinetic parameters obtained with the 3D FRET and SPR assays. We illustrate how 3D FRET has a vertical asymptote, and thus, is unable to capture the experimental features for lower values of the fundamental kinetic constants, *k*^+^ and *k*^−^.

We note that the comparison between the effective affinity constants of 2D FRET and AF assays, namely, 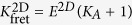 and 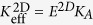, could be used to estimate *E*^2*D*^, a rotational diffusion constant common to all pMHC ligands in cell membranes for peptide ligands with *K*_*A*_ 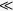 1.

Further, the comparison of the effective off-rate constants in 2D *versus* 3D FRET assays can be used to determine the 2D and 3D rotational diffusion constants as follows. First of all, we have already shown that 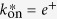 (Eq. (9)). Secondly, we have 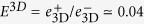
14. Therefore, 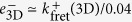. This result and the fact that 
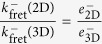
, yields 
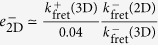
. And, finally, from 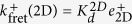 (see Eq. (15)) it follows 
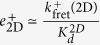
.

## Discussion

We have introduced and described a comprehensive mathematical framework, based on ordinary differential equations, that provides a unifying umbrella to describe and compare state-of-the-art experimental methods and also helps solve current experimental differences in the estimation of effective TCR-pMHC kinetic constants. Our study shows that much care must be taken when describing receptor-ligand interactions as a single step event, as this might not truly reflect the underlying complexity of these interactions, that involve both spatial processes (translational and rotational diffusion) and biochemical reactions.

Further, not only it reveals that what is called “on-” and “off-rate constants” often mean different things in different experimental assays, but also makes explicit their precise meaning in each assay. The same is true for a complex or a reaction product.

Moreover, in some cases the very concept of product cannot be defined due to limitations intrinsic to the experimental method. For example, in current FRET assays, being *close enough* and *properly oriented* cannot be distinguished from *being bound*. As we have shown here, the implications of this not only indicate that FRET experiments cannot yield estimations of on- and off-rate constants but help to properly interpret their results.

We believe our approach leads to two significant methodological improvements: on the one hand, making explicit the underlying hypothesis in a given experimental assay (and thus, in the experimentally determined kinetic constants), allows comparison with other assays and, on the other hand, theoretical models with finer *granularity* (more detailed steps) can help improve our understanding of how different molecular mechanisms contribute to receptor-ligand interactions, such as for example, mechanical versus chemical contributions^30^. Thus, splitting the binding step into two (or eventually, more) processes, such as actin recruiting and ligand-receptor binding, may help to decipher the role of mechanical forces in T cell activation.

Finally, we believe our analysis provides a clear route to identify the role of translational and rotational degrees of freedom in 2D and 3D experiments, and, for a fixed TCR, uniquely determines the relationship between the fundamental binding on- and off-rate constants and ligand potency.

## Additional Information

**How to cite this article:** Faro, J. *et al*. A unifying mathematical framework for experimental TCR-pMHC kinetic constants. *Sci. Rep.* **7**, 46741; doi: 10.1038/srep46741 (2017).

**Publisher's note:** Springer Nature remains neutral with regard to jurisdictional claims in published maps and institutional affiliations.

## Supplementary Material

Supplementary Information

## Figures and Tables

**Table 1:** Summary of the fundamental kinetic constants introduced in Eq. 1 and Fig. 2.

Step	Dimension	Parameter^†^	Units	Description	Refs
Diffusion	3D	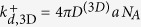	*M*^−1^ s^−1^	On-rate constant	16
Diffusion	3D	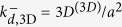	s^−1^	Off-rate constant	16
Diffusion	3D	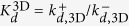	M^−1^	Affinity	16
Rotation	3D	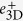	s^−1^	On-rate constant	15,21
Rotation	3D	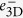	s^−1^	Off-rate constant	15,21
Rotation	3D	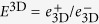	—	Affinity	15,21
Diffusion	2D	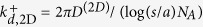	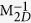 s^−1^	On-rate constant	16
Diffusion	2D	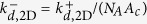	s^−1^	Off-rate constant	16
Diffusion	2D	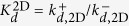	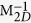	Affinity	16
Rotation	2D	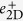	s^−1^	On-rate constant	15,21
Rotation	2D	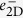	s^−1^	Off-rate constant	15,21
Rotation	2D	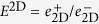	—	Affinity	15,21
Binding	—	*k*^+^	s^−1^	On-rate constant	15,21
Binding	—	*k*^−^	s^−1^	Off-rate constant	15,21
Binding	—	*K*_*A*_ = *k*^+^/*k*^−^	—	Affinity	15,21

Note that the fundamental binding constants (*k*^+^, *k*^−^) and the corresponding binding affinity constant (*K*_*A*_) are independent of the dimensionality of the experiment.

^†^*N*_*A*_, Avogradro constant. *A*_*c*_, effective cell surface area, defined as *A*_*c*_ = *πa*^2^, where *a* ≡ TCR encounter radius.

**Table 2:** Summary of the effective kinetic constants for the effective models described in Fig. 3 and their relationship to the fundamental kinetic constants of Table 1.

Model	effective constant	Eq. no.	Units	Dimension	Assay^†^ and references
PBA	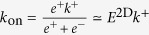	(6)	s^−1^	2D	TF^‡^ ref. 10
	*k*_off_ = *k*^−^	(7)	s^−1^	2D	TF^‡^ ref. 10
	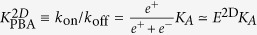	(8)	—	2D	TF^‡^ ref. 10
PBB	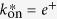	(9)	s^−1^	2D	TF^§^ ref. 10
	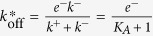	(10)	s^−1^	2D	TF^§^ ref. 10 and MD-SMFM ref. 25
	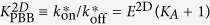	(11)	—	2D	TF^§^ ref. 10
	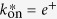	(12)	s^−1^	3D	Bulk FRET ref. 6
	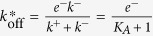	(13)	s^−1^	3D	Bulk FRET ref. 6 and MD-SMFM ref. 24
	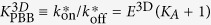	(14)	—	3D	Bulk FRET ref. 6
FRET	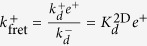	(15)	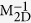 s^−1^	2D	SM-FRET ref. 11
	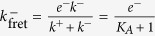	(16)	s^−1^	2D	SM-FRET ref. 11
	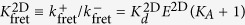	(17)	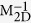	2D	SM-FRET ref. 11
SS	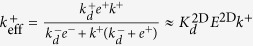	(18)	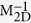 s^−1^	2D	AF ref. 10
	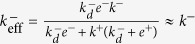	(19)	s^−1^	2D	AF ref. 10
	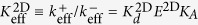	(20)	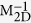	2D	AF ref. 10
	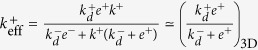	(21)	M^−1^s^−1^	3D	SPR refs 2,6,7
	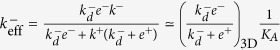	(22)	s^−1^	3D	SPR refs 2,6,7
	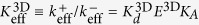	(23)	M^−1^	3D	SPR refs 2,6,7

PBA: Pre-binding model A; PBB: Pre-binding model B; FRET: FRET model; and SS: Single-step model.

^†^TF, thermal fluctuation. MD-SMFM, molecular diffusion and single molecule fluorescent microscopy. FRET, fluorescence resonance energy transfer. SM, single molecule. AF, adhesion frequency. SPR, surface plasmon resonance.

^‡^Assuming unbinding implies a change of orientation.

^§^Assuming multiple binding/unbinding events take place before a change of orientation.
