# Supplementary material for: A unifying mathematical framework for experimental TCR-pMHC kinetic constants
Source: Sci Rep. 2017 Apr 26;7:46741. doi: 10.1038/srep46741 (PMC5405415; doi:10.1038/srep46741)
Supplement: Supplementary Information [file srep46741-s1.pdf]

Supporting Information:
A unifying mathematical framework for experimental TCR-pMHC kinetic constants

Jose Faro, Mario Castro, Carmen Molina-París

20th February 2017

1 Description of the experimental systems

In this section we briefly describe the main 3D and 2D assays used by Zhu *et al.* [1] and Davis *et al.* [2], and identify the molecular states observed in the different experimental systems,as well as the associated effective kinetic constants.

1.1 3D assays

A) Surface plasmon resonance (SPR). Most procedures to analyze TCR-pMHC interactions in solution involve the use of SPR and either soluble TCR or pMHC as analyte (see [3] and references 3-7, 9-13, 15, 16, 18, 28, therein). In some publications, instead of using soluble TCR$\alpha\beta$ heterodimers (without the transmembrane domain), an engineered TRBV-TRAV single chain, composed of the variable domains of TCRα and TCRβ chains, was used as analyte [4–6]. In the SPR technique a specific molecule is linked to a chip surface within a chamber and an analyte in solution is allowed to flow over that surface in a laminar flow.

In this technique, a laser light beam, propagating through a prism and reaching a metal-coated dielectric interface, is perpendicularly polarized to the plane of the interface. At a critical incidence angle, this beam generates an evanescent wave, by internal reflection at the metal-coated surface, and is able to excite a non-radiative surface plasmon wave, which causes a sharp decrease in the angle-dependent intensity of the reflected light. This critical angle increases proportionally to the number of analyte molecules that bind to the molecules linked to the surface. Washing the chamber with a buffer solution without the analyte leads to a progressive detachment of the analyte and a corresponding decrease of the SPR critical incidence angle. The rates of increase and decrease of the critical angle are proportional to the binding and unbinding rate constants, respectively, of the analyte. Typically the interaction of the linked molecule and the analyte is assumed to be well described by a simple Langmuir model, namely,

$$A + B \underset{k_{\rm off}}{\overset{k_{\rm on}}{\rightleftarrows}} C,$$

where C is the bound complex of A and B.

B) Cells and fluorescent cell cytometer. Another technique to analyze TCR-pMHC interactions involves the use of OT-I T cells (expressing an ovalbumin peptide-specific

transgenic TCR) [7], or the human lymphoblastoid T2 cell line transfected with either L^d [8] or K^b [9] human MHC heavy chain. The OT-I and T2 cells were incubated for some time with saturating concentrations of soluble pMHC-phycoerythrin tetramers and TCR, respectively, washed and resuspended. T2-pMHC cells covered with TCR were incubated with an excess of an antibody anti-variable (V) region of the TCR, and at different times cells were incubated with biotinylated antibody anti-TCR+streptavidin-phycoerythrin, and the corresponding decrease of mean fluorescence intensity (MFI) with time was recorded. In the case of OT-I T cells covered with pMHC-phycoerythrin, these were incubated with a high concentration of an antibody anti-V region of the TCR, and the decrease of MFI with time was recorded. In each case the kinetics of MFI change was used to estimate the unbinding rate constant of TCR:pMHC complexes. This experimental procedure assumes that unbinding is essentially an irreversible process. This, however, may not be the case, particularly in the case of OT-I T cells and pMHC tetramers, where due to the tetravalent nature of the ligand, rebinding of a bound molecule through a previously unbound second or third site can still occur.

C) Bulk FRET in solution (3D FRET). In this technique specific pMHC and TCR molecules, labeled with donor and acceptor fluorochromes, respectively, are mixed in solution for FRET analysis in a small reaction chamber of a stopped-flow instrument [10]. When the TCR and the pMHC are close enough (and properly positioned) or even bound, the donor and acceptor are at an adequate distance to allow the generation of a FRET signal. The association rate constant can be estimated from the time course of the donor quenching due to FRET and assuming a two-step process, with an initial formation of a transient complex followed by a bound complex, that can be described by the following reactions:

$$A + B \underset{k_{\mathrm{off}}^{(1)}}{\overset{k_{\mathrm{on}}^{(1)}}{\rightleftarrows}} A :: B \underset{k_{\mathrm{off}}^{(2)}}{\overset{k_{\mathrm{on}}^{(2)}}{\rightleftarrows}} C,$$

where C is the bound complex of A and B. However, in a previous study by the same group using SPR, this two-step process was deemed incapable to explain the observed experimental results [11]. This experimental design cannot distinguish between bound molecules and those close enough and properly oriented. Therefore, the assumed two-step process should correspond to: (1) free chemical species, (2) encounter complexes, and (3) a combination of oriented and bound complexes.

D) Single molecule fluorescent microscopy (SMFM) [12]. This assay is devised to measure the unbinding rate constant of TCR-pMHC complexes. It makes use of T cells attached to a poly-lysine-coated borosilicate slide and quantum-dot-tagged pMHC monomers added in solution. Using fluorescent microscopy, bound quantum-dot-pMHC molecules were detected as single fluorescent spots on the T cell membrane. The distribution of the duration of individual pMHC binding events, starting and ending in a fixed time window, was used to calculate the average dwell-time and from it the dissociation rate constant.

A reported 2D-variant of this assay makes use of the duration of individual fluorescent spots of only those fluorochrome-tagged pMHC molecules showing slow diffusion, which characterizes TCR-bound pMHC molecules [13].

1.2 2D assays

A) Adhesion frequency assay [1]. This assay makes use of a red blood cell (RBC) decorated with univalent pMHC molecules or an RBC with an attached bead decorated with pMHC. With the help of a micropipette, a T cell was positioned to touch the RBC or the bead. After a given time, RBC elongation or bead displacement on T cell retraction, due to binding, may or may not happen. For a given contact time, many trials are performed and the frequency of binding events is recorded. This procedure is followed for a range of contact times. Assuming a simple Langmuir model and based on a probabilistic model for adhesion kinetics mediated by a small number of bonds, the corresponding affinity constant and dissociation rate constant can be derived by a best-fit procedure.

B) Thermal fluctuation assay [1]. This assay is based on the reduction/resumption of thermal position fluctuations of a pMHC-decorated bead touching a TCR-transgenic T cell held in a fixed position. The reduction of the bead's fluctuation is caused by bond formation between one or more bead pMHCs and T cell TCRs, restricting the movements of the bead. Time periods of restricted movement, t_b, are considered durations of the bound state. Fitting the frequency distribution of many recorded t_b periods to an exponential function allows one to derive an dissociation rate constant for the reaction.

C) FRET on cell membrane (2D FRET) [2]. This experimental setting makes use of TCR-transgenic T cells with the V-regions stained with a donor fluorochrome, MHC molecules complexed to peptides tagged with an acceptor fluorochrome, and total internal reflection microscopy to allow single molecule FRET. As is the case in the 3D assay, when the TCR and pMHC are bound or close to each other and oriented, the donor and acceptor dyes are at a suitable distance that leads to the generation of a FRET signal. The duration in single synapses of FRET signals that started and finished during an experimental recording was considered to correspond to a bound time period. Measuring the duration of many binding events and fitting their frequency distribution to an exponential function allows to obtain a dissociation rate constant for the reaction. In addition, after a long enough synaptic period ($\leq$ 2.5 min), to safely assume a steady state has been reached, the local density of total TCRs, pMHCs and TCR-pMHC complexes was determined in a single synapse, considering average intensities of single-molecule fluorescence. This allowed to estimate the average local effective 2D affinity constant (K_a), and from this value and the dissociation rate constant, the association rate constant can be estimated.

1.3 Mathematical description

In the main text, the following set of coupled ordinary differential equations (ODEs) are derived from the general reaction scheme, in terms of the fundamental kinetic constants

[see Eq. (1) and Eqs. (2)-(5) therein],

$$\frac{d[R]}{dt} = \frac{d[L]}{dt} = -k_d^+ \, [R] \cdot [L] + k_d^- \, [RL^*], \quad (1)$$

$$\frac{d[RL^*]}{dt} = k_d^+ \, [R] \cdot [L] - k_d^- \, [RL^*] - e^+ \, [RL^*] + e^- \, [RL], \quad (2)$$

$$\frac{d[RL]}{dt} = e^+ \, [RL^*] - e^- \, [RL] - k^+ \, [RL] + k^- \, [C], \quad (3)$$

$$\frac{d[C]}{dt} = k^+ \, [RL] - k^- \, [C]. \quad (4)$$

In a similar fashion, we can obtain a specific set of coupled ODEs for every experimental model considered in Fig. 3 of the main text. Thus,

- **Pre-binding model A**:

$$\frac{d[R]}{dt} = \frac{d[L]}{dt} = -k_d^+ \, [R] \cdot [L] + k_*^- \, \langle RL \rangle, \quad (5)$$

$$\frac{d\langle RL \rangle}{dt} = k_d^+ \, [R] \cdot [L] - k_*^- \, \langle RL \rangle - k_{\text{on}} \, \langle RL \rangle + k_{\text{off}} \, [C], \quad (6)$$

$$\frac{d[C]}{dt} = k_{\text{on}} \, \langle RL \rangle - k_{\text{off}} \, [C]. \quad (7)$$

- **Pre-binding model B**:

$$\frac{d[R]}{dt} = \frac{d[L]}{dt} = -k_d^+ \, [R] \cdot [L] + k_d^- \, [RL^*], \quad (8)$$

$$\frac{d[RL^*]}{dt} = k_d^+ \, [R] \cdot [L] - k_d^- \, [RL^*] - k_{\text{on}}^* \, [RL^*] + k_{\text{off}}^* \, \langle C \rangle, \quad (9)$$

$$\frac{d\langle C \rangle}{dt} = k_{\text{on}}^* \, [RL^*] - k_{\text{off}}^* \, \langle C \rangle. \quad (10)$$

- **FRET model:**

$$\frac{d\langle R \rangle}{dt} = \frac{d\langle L \rangle}{dt} = -k_{\text{fret}}^+ \, \langle R \rangle \cdot \langle L \rangle + k_{\text{fret}}^- \, \langle C \rangle, \quad (11)$$

$$\frac{d\langle C \rangle}{dt} = k_{\text{fret}}^+ \, \langle R \rangle \cdot \langle L \rangle - k_{\text{fret}}^- \, \langle C \rangle. \quad (12)$$

- **Single-step model:**

$$\frac{d[R]}{dt} = \frac{d[L]}{dt} = -k_{\text{eff}}^+ \, [R] \cdot [L] + k_{\text{eff}}^- [C], \quad (13)$$

$$\frac{d[C]}{dt} = k_{\text{eff}}^+ \, [R] \cdot [L] - k_{\text{eff}}^- [C]. \quad (14)$$

With these equations at hand, we can proceed to find a relationship between the fundamental kinetic rate constants of the general model and the effective kinetic constants in the coarse-grained experimental descriptions.

2 Effective kinetic constants in terms of fundamental ones

2.1 Local steady state balance

In order to derive the simplified equations of pre-binding models A and B, Eqs. (5)-(10), from the general model described by Eqs. (1)-(4), we introduce an additional hypothesis: due to experimental limitations, it might not be possible to discriminate between the different molecular states. In particular, when orientation prior to binding occurs fast enough, we cannot distinguish between encounter-non-oriented and encounter-oriented states. In mathematical terms, this means that, in a coarse-grained observation timescale, we can safely assume that [1]:

$$e^+ \, [RL^*] = e^- \, [RL], \tag{15}$$

for the pre-binding model A, and

$$k^+ \, [RL] = k^- \, [C] \tag{16}$$

for the pre-binding model B.

We first carry out model reduction for the pre-binding model A. Defining $\langle RL \rangle = [RL^*] + [RL]$ and using Eq. (15) one gets:

$$[RL^*] = \frac{\langle RL \rangle}{1 + e^+/e^-}, \qquad [RL] = \frac{\langle RL \rangle}{1 + e^-/e^+}.$$

We add Eqs. (2)-(3) and substitute $[RL^*]$ and $[RL]$ in the resulting equation, as well as in Eq. (4) to obtain:

$$\frac{d[R]}{dt} = \frac{d[L]}{dt} = -k_d^+ \, [R] \cdot [L] + \frac{k_d^- \, e^-}{e^+ + e^-} \, \langle RL \rangle, \tag{17}$$

$$\frac{d\langle RL \rangle}{dt} = k_d^+ \, [R] \cdot [L] - \left(\frac{k_d^- \, e^- + e^+ \, k^+}{e^+ + e^-} \right) \langle RL \rangle + k^- \, [C], \tag{18}$$

$$\frac{d[C]}{dt} = \frac{e^+ \, k^+}{e^+ + e^-} \, \langle RL \rangle - k^- \, [C]. \tag{19}$$

Equations (18)-(19) allow the identification of the effective rate constants in Eqs. (6)-(7) in terms of the fundamental rate constants. In particular, from the constants multiplying $\langle RL \rangle$ and $[C]$ in Eqs. (19) and (7) we find,

$$k_{\text{on}} = \frac{e^+ \, k^+}{e^+ + e^-}, \qquad k_{\text{off}} = k^-. \tag{20}$$

These values correspond to Eqs. (3)-(5) in Table 2 of the main text. Using Eqs. (17) and (5), we could determine k_*^-, so that

$$k_*^- = \frac{k_d^- \, e^-}{e^+ + e^-}. \tag{21}$$

[1]In statistical thermodynamics this hypothesis is often referred to as local equilibrium hypothesis. We prefer to use the term steady state to avoid the connotation the word "equilibrium" is associated with in biochemistry.

In a similar way and for the pre-binding model B, we define $\langle C \rangle = [RL] + [C]$, and make use of Eq. (16) to obtain $[RL]$ in terms of $\langle C \rangle$. We add Eqs. (3)-(4) and substitute $[RL]$ to obtain:

$$\frac{d[RL^*]}{dt} = k_d^+ [R] \cdot [L] - k_d^- [RL^*] - e^+ [RL^*] + \frac{e^- k^-}{k^+ + k^-} \langle C \rangle, \tag{22}$$

$$\frac{d\langle C \rangle}{dt} = e^+ [RL^*] - \frac{e^- k^-}{k^+ + k^-} \langle C \rangle. \tag{23}$$

As we have done above, Eqs. (22)-(23) allow us to identify the effective rate constants of Eqs. (9)-(10) in terms of the fundamental ones

$$k_{\text{on}}^* = e^+, \qquad k_{\text{off}}^* = \frac{e^- k^-}{k^+ + k^-}. \tag{24}$$

These equations correspond to Eqs. (6)-(8) in Table 2 of the main text.

2.2 Local steady state balance and perturbation analysis

The method introduced in the preceding section cannot be directly applied to the FRET model, as it involves the meta-states $\langle R \rangle$, $\langle L \rangle$ and $\langle C \rangle$, and the corresponding concentrations $[\langle R \rangle]$ and $[\langle L \rangle]$ enter non-linearly in the effective reactions. To solve this problem we proceed in two steps.

First, we assume, a local steady state hypothesis:

$$k_d^+ [R] \cdot [L] = k_d^- [RL^*], \tag{25}$$

$$k^+ [RL] = k^- [C]. \tag{26}$$

and follow the same procedure as above. Specifically, we define $\langle R \rangle = [R] + [RL^*]$, $\langle L \rangle = [L] + [RL^*]$ and $\langle C \rangle = [RL] + [C]$ and add Eqs. (1)-(2) to obtain an equation for the time evolution of $\langle R \rangle$. In the same way as for $\langle R \rangle$, a differential equation can be obtained for the remaining meta-states $\langle L \rangle$ and $\langle C \rangle$, namely:

$$\frac{d\langle R \rangle}{dt} = \frac{d\langle L \rangle}{dt} = -e^+ [RL^*] + e^- [RL], \tag{27}$$

$$\frac{d\langle C \rangle}{dt} = e^+ [RL^*] - e^- [RL]. \tag{28}$$

We now need to obtain expressions for $[RL^*]$ and $[RL]$ in terms of the meta-states $\langle L \rangle$ and $\langle C \rangle$. We make use of Eqs. (25) and (26) to find

$$[RL^*] = \frac{k_d^+}{k_d^-} (\langle R \rangle - [RL^*]) \cdot (\langle L \rangle - [RL^*]), \tag{29}$$

$$[RL] = \frac{k^-}{k^+ + k^-} \langle C \rangle. \tag{30}$$

Eqn. (29) is quadratic and can be solved explicitly. It has two solutions that involve a square root due to the quadratic nature of the equation. Thus, the exact solution does not allow to obtain a linear relationship between the effective and fundamental kinetic rates. To overcome this limitation, we first rewrite Eq. (29) in terms of number

concentrations instead of molar concentrations. Namely, we define n_{RL^*}, n_R and n_L as the absolute number of molecular species RL^*, $\langle R\rangle$ and $\langle L\rangle$, respectively. In general, $n_X = [X]\ N_A\ V$ where V is the experimental volume and N_A is Avogadro constant. Thus, Eq. (29) can be written as:

$$n_{RL^*} = \frac{k_d^+}{k_d^- N_A\ V}(n_R - n_{RL^*}) \cdot (n_L - n_{RL^*}). \tag{31}$$

Now, we can take advantage of the following relationship between the diffusion rate constants [14–16]:

$$\frac{k_d^+}{k_d^-\ N_A\ V} = \frac{N}{\pi}\left(\frac{s}{a}\right)^d, \tag{32}$$

where N is the number of receptors, $d = 2, 3$ for 2D and 3D systems, respectively, and s and a are the approximate diameters of the receptor and the cell, respectively. As this ratio is small for the receptors and cells under consideration, we can perform a singular perturbation analysis of Eq. (31) in terms of a small parameter $\epsilon = k_d^+/(k_d^-\ N_A\ V)$. Note that $\epsilon \ll 1$ as s is about ten nanometers and a a few microns, so that $s/a \sim 10^{-2}$. The number of receptors is of the order of 10^4 in the 3D case and around 10^2 in the 2D case (the synapse) so, effectively, in both cases $\epsilon \sim 10^{-2}$.

We now write Eq. (31) as an iterative relation for an unknown $x \equiv n_{RL^*}$ for different orders i in the approximation. Namely,

$$x_{i+1} = \epsilon\ (n_R - x_i)(n_L - x_i).$$

To lowest order we have, $x_0 = \epsilon\ n_R\ n_L$; that is

$$[RL^*] \approx \frac{k_d^+}{k_d^-}\ \langle R\rangle \cdot \langle L\rangle. \tag{33}$$

The next order leads to

$$x_1 = \epsilon\ (n_R - \epsilon n_R n_L)(n_L - \epsilon n_R n_L) = x_0 - \epsilon\ x_0(n_R + n_L) + \epsilon\ x_0^2.$$

For the sake of simplicity, we only make use of the lowest order, as higher corrections are $O(\epsilon)$, and as mentioned above $\epsilon \sim 10^{-2}$.

We then substitute $[RL]$ and $[RL^*]$, as defined, respectively, by Eqs. (30) and (33), into Eqs. (27)-(28) to obtain:

$$\begin{aligned} \frac{d\langle R\rangle}{dt} = \frac{d\langle L\rangle}{dt} &= -\frac{e^+\ k_d^+}{k_d^-}\ \langle R\rangle \cdot \langle L\rangle + \frac{e^-\ k^-}{k^+ + k^-}\ \langle C\rangle, \\ \frac{d\langle C\rangle}{dt} &= \frac{e^+\ k_d^+}{k_d^-}\ \langle R\rangle \cdot \langle L\rangle - \frac{e^-\ k^-}{k^+ + k^-}\ \langle C\rangle. \end{aligned}$$

We can conclude that

$$k_{\text{fret}}^+ = \frac{e^+\ k_d^+}{k_d^-}, \tag{34}$$

$$k_{\text{fret}}^- = \frac{e^-\ k^-}{k^+ + k^-}. \tag{35}$$

2.3 The quasi-steady state approximation

We conclude our mathematical analysis of the different experimental systems, with the study of the single-step model. In this case, the intermediate states $[RL^*]$ and $[RL]$ (encounter and oriented configurations, respectively) are assumed to be transient. This implies that the timescales associated to these states are short enough, so we can consider them to be *slaved* to the concentration of free species ($[R]$ and $[L]$) and the bound complex ($[C]$). This approximation, often referred to as the quasi-steady state approximation (QSSA) is widely used, not only due to its simplicity, but as it has proven to be a good approximation [17], and it has served as a starting point for improved approximations [18].

In our case, the use of this approximation means dropping the time derivatives in Eqs. (2)-(3) and simplifying the *slaved* concentrations in terms of the remaining ones:

$$[RL^*] = \frac{k_d^+ \, (e^- + k^+)}{e^- \, k_d^- + k^+ \, (e^+ + k_d^-)} \, [R] \cdot [L] + \frac{e^- \, k^-}{e^- \, k_d^- + k^+ \, (e^+ + k_d^-)} \, [C], \quad (36)$$

$$[RL] = \frac{e^+ \, k_d^+}{e^- \, k_d^- + k^+ \, (e^+ + k_d^-)} \, [R] \cdot [L] + \frac{k^- \, (e^+ + k_d^-)}{e^- \, k_d^- + k^+ \, (e^+ + k_d^-)} \, [C]. \quad (37)$$

Finally, substituting $[RL^*]$ and $[RL]$, as defined above, into Eqs. (1) and (4) leads to:

$$\begin{aligned} \frac{d[R]}{dt} &= \frac{d[L]}{dt} = -\frac{k_d^+ \, e^+ \, k^+}{k_d^- \, e^- + k^+ \, (k_d^- + e^+)} \, [R] \cdot [L] + \frac{k_d^- \, e^- \, k^-}{k_d^- \, e^- + k^+ \, (k_d^- + e^+)} \, [C], \\ \frac{d[C]}{dt} &= \frac{k_d^+ \, e^+ \, k^+}{k_d^- \, e^- + k^+ \, (k_d^- + e^+)} \, [R] \cdot [L] - \frac{k_d^- \, e^- \, k^-}{k_d^- \, e^- + k^+ \, (k_d^- + e^+)} [C]. \end{aligned}$$

If we replace the ligand concentration, $[L]$, by the ligand number of molecules, $n_L = N_A \, V \, [L]$, we obtain a relation for $k_{\text{eff}}^+/(N_A \, V)$ instead of k_{eff}^+.

We conclude with the expression of the effective rate constants in terms of the fundamental ones for the single-step model:

$$k_{\text{eff}}^+ = \frac{k_d^+ \, e^+ \, k^+}{k_d^- \, e^- + k^+ \, (k_d^- + e^+)}, \quad (38)$$

$$k_{\text{eff}}^- = \frac{k_d^- \, e^- \, k^-}{k_d^- \, e^- + k^+ \, (k_d^- + e^+)}. \quad (39)$$

References

[1] Huang, J. *et al.* The kinetics of two dimensional TCR and pMHC interactions determine T cell responsiveness. *Nature* **464**, 932; DOI:10.1038/nature08944 (2010).

[2] Huppa, J. *et al.* TCR–peptide–MHC interactions in situ show accelerated kinetics and increased affinity. *Nature* **463**, 963–967; DOI:10.1038/nature08746 (2010).

[3] Stone, J. D., Chervin, A. S. & Kranz, D. M. T-cell receptor binding affinities and kinetics: impact on T-cell activity and specificity. *Immunology* **126**, 165–176; DOI:10.1111/j.1365-2567.2008.03015.x (2009).

[4] Garcia, K. C., Radu, C. G., Ho, J., Ober, R. J. & Ward, E. S. Kinetics and thermodynamics of T cell receptor- autoantigen interactions in murine experimental autoimmune encephalomyelitis. *Proceedings of the National Academy of Sciences of the United States of America* **98**, 6818–6823; DOI:10.1073/pnas.111161198 (2001).

[5] Weber, K. S., Donermeyer, D. L., Allen, P. M. & Kranz, D. M. Class II-restricted T cell receptor engineered in vitro for higher affinity retains peptide specificity and function. *Proceedings of the National Academy of Sciences of the United States of America* **102**, 19033–19038; DOI:10.1073/pnas.0507554102 (2005).

[6] Jones, L. L., Colf, L. A., Stone, J. D., Garcia, K. C. & Kranz, D. M. Distinct CDR3 conformations in TCRs determine the level of cross-reactivity for diverse antigens, but not the docking orientation. *Journal of immunology (Baltimore, Md. : 1950)* **181**, 6255–6264; DOI:10.4049/jimmunol.181.9.6255 (2008).

[7] Rosette, C. *et al.* The impact of duration versus extent of TCR occupancy on T cell activation: a revision of the kinetic proofreading model. *Immunity* **15**, 59–70; DOI:10.1016/S1074-7613(01)00173-X (2001).

[8] Holler, P. D., Lim, A. R., Cho, B. K., Rund, L. A. & Kranz, D. M. CD8-T cell transfectants that express a high affinity T cell receptor exhibit enhanced peptide-dependent activation. *The Journal of experimental medicine* **194**, 1043–1052; DOI:10.1084/jem.194.8.1043 (2001).

[9] Holler, P. D., Chlewicki, L. K. & Kranz, D. M. TCRs with high affinity for foreign pMHC show self-reactivity. *Nature immunology* **4**, 55–62; DOI:10.1038/ni863 (2003).

[10] Gakamsky, D. M. *et al.* Kinetic evidence for a ligand-binding-induced conformational transition in the T cell receptor. *Proceedings of the National Academy of Sciences of the United States of America* **104**, 16639–16644; DOI:10.1073/pnas.0707061104 (2007).

[11] Gakamsky, D. M., Luescher, I. F. & Pecht, I. T cell receptor-ligand interactions: a conformational preequilibrium or an induced fit. *Proceedings of the National Academy of Sciences of the United States of America* **101**, 9063–9066; DOI:10.1073/pnas.0402840101 (2004).

[12] Stepanek, O. *et al.* Coreceptor scanning by the T cell receptor provides a mechanism for T cell tolerance. *Cell* **159**, 333–345; DOI:10.1016/j.cell.2014.08.042 (2014).

[13] O'Donoghue, G. P., Pielak, R. M., Smoligovets, A. A., Lin, J. J. & Groves, J. T. Direct single molecule measurement of TCR triggering by agonist pMHC in living primary T cells. *Elife* **2**, e00778; DOI:10.7554/eLife.00778 (2013).

[14] Lauffenburger, D. & Linderman, J. *Receptors: models for binding, trafficking, and signaling* (Oxford University Press, USA, 1996).

[15] Delisi, C. The effect of cell size and receptor density on ligand-receptor reaction rate constants. *Molecular immunology* **18**, 507–511; DOI:10.1016/0161-5890(81)90128-0 (1981).

[16] DeLisi, C. & Wiegel, F. W. Effect of nonspecific forces and finite receptor number on rate constants of ligand–cell bound-receptor interactions. *Proceedings of the National Academy of Sciences* **78**, 5569–5572; DOI:http://www.pnas.org/content/78/9/5569.short (1981).

[17] Segel, L. A. & Slemrod, M. The Quasi-Steady-State Assumption: a case study in perturbation. *SIAM review* **31**, 446–477; DOI:10.1137/1031091 (1989).

[18] Borghans, J. A., De Boer, R. J. & Segel, L. A. Extending the Quasi-Steady State Approximation by changing variables. *Bulletin of mathematical biology* **58**, 43–63; DOI:10.1016/0092-8240(95)00306-1 (1996).
